# Vitamin D deficiency among smear positive pulmonary tuberculosis patients and their tuberculosis negative household contacts in Northwest Ethiopia: a case–control study

**DOI:** 10.1186/s12941-017-0211-3

**Published:** 2017-05-11

**Authors:** Belay Tessema, Feleke Moges, Dereje Habte, Nebiyu Hiruy, Shewaye Yismaw, Kassahun Melkieneh, Yewulsew Kassie, Belaineh Girma, Muluken Melese, Pedro G. Suarez

**Affiliations:** 1 Department of Medical Microbiology, College of Medicine and Health Sciences, University of Gondar, Gondar, Ethiopia; 2 Management Sciences for Health, Help Ethiopia Address the Low Performance of Tuberculosis (HEAL TB) Project, Addis Ababa, Ethiopia; 3 Department of Chemistry, College of Natural and Computational Sciences, University of Gondar, Gondar, Ethiopia; 4 USAID/Ethiopia, Addis Ababa, Ethiopia; 5 Monitoring and Evaluation TA, National Tuberculosis Program, Lilongwe, Malawi; 6 Management Sciences for Health, Health Programs Group, Arlington, VA USA

**Keywords:** Vitamin D deficiency, Tuberculosis, Household contacts

## Abstract

**Background:**

Vitamin D is a fat-soluble vitamin that increases the immunity against tuberculosis (TB), decreases the re-activation of latent TB and reduces the severity of active TB disease. Epidemiological studies on the prevalence of vitamin D deficiency, and its association with TB showed inconsistent results in different countries. This study was aimed to determine the prevalence of vitamin D deficiency and its association with TB in Northwest Ethiopia.

**Methods:**

A case–control study was conducted among smear positive pulmonary tuberculosis patients and their household contacts without symptoms suggestive of TB. Study participants were recruited at 11 TB diagnostic health facilities in North and South Gondar zones of Amhara region between May 2013 and April 2015. The spot-morning-spot sputum samples and 5 ml blood sample were collected prior to commencing TB treatment for the diagnosis of TB and serum vitamin D assay, respectively. The diagnosis of TB was performed using smear microscopy and GeneXpert. Serum vitamin D level was analyzed using VIDAS 25 OH Vitamin D Total testing kits (Biomerieux, Marcy I’Etoile, France) on mini VIDAS automated immunoassay platform. Vitamin D status was interpreted as deficient (<20 ng/ml), insufficient (20–29 ng/ml), sufficient (30–100 ng/ml) and potential toxicity (>100 ng/ml).

**Results:**

Of the total study participants, 134 (46.2%) were vitamin D deficient, and only 56 (19.3%) had sufficient vitamin D level. A total of 59 (61.5%) TB patients and 75 (38.7%) non TB controls were vitamin D deficient. Results of multivariate logistic regression analyses showed a significantly higher vitamin D deficiency among tuberculosis cases (p < 0.001), females (p = 0.002), and urban residents (p < 0.001) than their respective comparison groups. Moreover, age groups of 35–44 (p = 0.001), 45–54 (p = 0.003) and ≥55 (p = 0.001) years had significantly higher vitamin D deficiency compared with age group <15 years.

**Conclusions:**

Vitamin D deficiency is highly prevalent among TB patients and non TB controls in Ethiopia where there is year round abundant sunshine. Study participants with tuberculosis, females, older age groups, and urban residents had significantly higher prevalence of vitamin D deficiency. These findings warrant further studies to investigate the role of vitamin D supplementation in the prevention and treatment of tuberculosis in high TB burden countries like Ethiopia.

## Background

Tuberculosis (TB) remains one of the deadliest communicable diseases in the world. According to World Health Organization (WHO) global TB report 2014, Ethiopia ranked 7th among the 22 high TB burden countries and 15th among the 27 high Multi-Drug Resistant Tuberculosis (MDR-TB) burden countries in the world. Ethiopia had an estimated 211 prevalent TB case per 100,000 population and a total of 30,000 TB related deaths. Among patients with notified pulmonary TB cases in the year 2013, there was an estimated 1400 MDR-TB cases in the country [1].

Vitamin D is a fat-soluble vitamin that plays important role against infectious diseases including tuberculosis [2]. The two most likely ways by which vitamin D controls the immune system in the fight against *M. tuberculosis* are: (1) Vitamin D decreases the viability of *M. tuberculosis* by increasing the fusion of the phagosome and lysosome in infected macrophages [3]; (2) It may improve the production of LL-37, an antimicrobial peptide of the cathelicidin family [3–6]. Defensin and cathelicidin are some of the antimicrobial peptides that involve as a first line of defenses in the inhibition of infections with infectious diseases such as TB. The vitamin D in neutrophils and macrophages controls the hCAP-18 gene that codes for LL-37, hence, vitamin D may increase the host body defenses to control TB [3–7]. The use of vitamin D to treat TB patients has a long history even before Robert Koch discovered the etiologic agent of TB [3].

Vitamin D can be present naturally in very few foods, as dietary supplements, and produced endogenously when ultraviolet rays from sunlight strike the skin and trigger vitamin D synthesis, however, vitamin D deficiency has been shown to be common in low-income countries, including those in equatorial Africa [8–10].

Inadequate vitamin D level, vitamin D insufficiency or deficiency, is a global problem. It was estimated that one billion people globally have inadequate level of vitamin D [11]. Previous studies have shown that vitamin D deficiency is a problem in Africa [12–16]. Higher prevalence of vitamin D deficiency was observed among untreated pulmonary TB patients compared to the non-TB healthy controls [17]. Vitamin D deficiency among TB patients have been reported in different African countries with the prevalence ranging from 8.5 to 62.7% [8, 10, 14–16]. A study conducted in the central part of Ethiopia showed a prevalence of 42% vitamin D deficiency among school children [18]. Another study conducted in Israel among adult Ethiopian women immigrants showed that all women (five) with hypocalcaemia were also vitamin D deficient [19].

Previous reports showed that inadequate vitamin D status is a public health problem globally. However, there are discrepancies in the prevalence of vitamin D deficiency, and the association between vitamin D deficiency and TB among studies conducted in different countries. The lack of consistency of results may be due to the fact that the level of vitamin D in human is affected by several factors such as race, latitude, exposure to sunlight, socioeconomic status, nutrition, and traditional/cultural traits [20]. As far as our knowledge is concerned, there are quite few reports, one on the prevalence of vitamin D deficiency among children and the other on Ethiopian women immigrants, but no data on the association between vitamin D deficiency and tuberculosis in Ethiopia. Therefore, this study was aimed to determine the prevalence of vitamin D deficiency and the association between vitamin D deficiency and tuberculosis among smear positive pulmonary tuberculosis patients and their household contacts without symptoms suggestive of TB in Northwest Ethiopia.

## Methods

### Study design, settings and study period

A prospective case–control study was conducted among smear positive pulmonary tuberculosis patients and their household contacts without symptoms suggestive of TB (controls). A total of 290 study participants were included in this study. Of them, 96 participants are TB patients and 194 are non TB controls. Study participants were recruited at 11 TB diagnostic health facilities in North and South Gondar zones of Amhara region between May 2013 and April 2015. The 11 TB diagnostic health facilities included in this study are Gondar Health Center (HC), Marakie HC, Woleka HC, Gebriel HC, Azezo HC, Kola Duba HC, Tseda HC, Maksegnit HC, Enferanze HC, Addis Zemen HC, and Woreta HC.

Based on the 2007 census conducted by the central statistical agency of Ethiopia (CSA) [21], Amhara region has a total population of 17,221,976, of whom, 8,641,580 are males and 8,580,396 are females. It is the second populous region in the country. The Amhara region extends from 9° to 13° 45′ N and 36° to 40° 30′ E. It covers approximately 161,828.4 sq km in area. This is 11% of Ethiopia’s total area. This land consists of three major geographical zones. These are highlands (above 2300 m above sea level), semi-highlands (1500–2300 m above sea level) and lowlands (below 1500 m above sea level) accounting 20, 44 and 28%, respectively. The Amhara region, like the rest of the country, is located within the tropics where there is no significant variation in day length and the angle of the sun throughout the year. As a result, the average annual temperatures in the region are high. The region has three climatic zones such as hot dry tropical (800–1830 m above sea level), sub tropical (1830–2440 m above sea level), cool temperature (over 2440 m above sea level) with average annual temperatures 27, 22 and 16 °C, respectively. In all climatic zones there is sunshine throughout the year.

### Recruitment of the study participants

All smear positive TB patients diagnosed during the study period and their household contacts without symptoms suggestive of TB, volunteered to participate were enrolled in this study. Clinical screening of contacts was conducted using the WHO screening criteria [22] in 2 weeks time after the index case diagnosed. For all study subjects, information on the socio-demographic data was collected using structured questionnaire. The spot-morning-spot sputum samples and 5 ml venous blood sample were collected prior to commencing TB treatment. Sputum and serum specimens were stored at −20 °C until transported to University of Gondar and Felege Hiwot hospitals using cold box. Sputum samples transported to University of Gondar Hospital for GeneXpert testing while serum samples transported to Felege Hiwot Hospital, Bahir Dar for serum Vitamin D assay.

### Laboratory diagnosis of tuberculosis

The spot- morning-spot sputum samples collected from presumptive TB patients were examined using either Zihel Neelsen microscopy or light emitting diode (LED) fluorescence microscopy (FM) for acid fast bacilli (AFB) at respective health facilities following the manufacturer’s procedures (Zeiss, Germany). Split sputum samples of all smear positive TB patients were further examined using the Gene Xpert MTB/RIF (Cepheid, USA) following the standard procedure to confirm TB positive study participants.

Study participants were considered TB positive, if their sputum samples are positive for TB by GeneXpert or both GeneXpert and smear microscopy. Study subjects were considered TB negative controls, if household contacts of smear positive TB cases had no symptoms suggestive of TB. TB negative study participants were included in this study as a control to compare the association between TB and vitamin D deficiency.

### Measurement of serum vitamin D concentration

Serum samples were separated by centrifugation and frozen immediately at −20 °C. Serum 25 OH Vitamin D levels were measured using a VIDAS 25 OH Vitamin D Total testing kits (Biomerieux, Marcy I’Etoile, France) on mini VIDAS automated immunoassay platform. VIDAS 25 OH Vitamin D Total is a quantitative test using Enzyme Linked Fluorescent Assay (ELFA) technology. The vitamin D status of study participants was interpreted based on the serum 25-(OH) Vitamin D concentration following the manufacturer’s instructions as deficient (<20 ng/ml), insufficient (20–29 ng/ml), sufficient (30–100 ng/ml) and potential toxicity (>100 ng/ml). The VIDAS 25-OH Vitamin D Total assay showed excellent performance with correlation of r = 0.93 compared with the reference standard liquid chromatography/mass spectrometry methods (LC–MS/MS) [23].

### Quality control of laboratory methods

The reliability of the study findings was guaranteed by implementing quality control measures throughout the whole process of the laboratory work. All materials, equipment and procedures were adequately controlled.

### Statistical analysis

The data analysis was made using SPSS version 16 software (SPSS Inc., Chicago, IL) after the data was entered and properly cleared. The Chi square test was used to compare the categorical variables. The two important outcome variables assessed using logistic regression analysis model were TB and Vitamin D deficiency. The odds ratios (OR) and 95% confidence intervals (CI) were calculated for demographic and epidemiologic variables by using logistic regression analysis. A multivariate binary logistic regression analysis was used to identify independent risk factors associated with TB and vitamin D deficiency in the study participants. *P* value <0.05 was considered statistically significant.

## Results

### General characteristics of study participants

A total of 290 study participants (141 males and 149 females) were included in this study. The study included 96 TB patients (57 males and 39 females) and 194 non TB controls (84 males and 110 females). Majority, 180 (62.1%) of the study participants were urban residents. Eighty one of the study participants (27.9%) were children, <15 years of age. The proportion of children among controls was 35.1% compared to the 13.5% children among TB cases. The mean age (SD) of the study subjects was 27.1 (16.7) years (Table 1).

**Table 1:** Socio-demographic and clinical characteristics of study participants (N = 290)

Characteristics	TB patients (N = 96) N (%)	Non TB controls (N = 194) N (%)	Total (N = 290)
Gender			
Male	57 (59.4)	84 (43.3)	141 (48.6)
Female	39 (40.6)	110 (56.7)	149 (51.4)
Residence			
Rural	34 (35.4)	76 (39.2)	110 (37.9)
Urban	62 (64.6)	118 (60.8)	180 (62.1)
Age group in years			
<15	13 (13.5)	68 (35.1)	81 (27.9)
15–24	27 (28.1)	33 (17.0)	60 (20.7)
25–34	30 (31.2)	33 (17.0)	63 (21.7)
35–44	10 (10.4)	27 (13.9)	37 (12.8)
45–54	6 (6.2)	17 (8.8)	23 (7.9)
55+	10 (10.4)	16 (8.2)	26 (9.0)

N number, *TB* tuberculosis

### Serum vitamin D levels

Of the total study participants, 134 (46.2%) were vitamin D deficient, 100 (34.5%) had insufficient vitamin D and only 56 (19.3%) study participants had sufficient vitamin D level. In the TB positive cases, 59 patients (61.5%) were vitamin D deficient and 20 (20.8%) had insufficient vitamin D. While, 75 study participants (38.7%) in the non TB controls were vitamin D deficient and 80 (41.2%) had insufficient vitamin D (Table 2).

**Table 2:** Vitamin D levels among study participants (n = 290)

Characteristics	Vitamin D levels			Total N
	Deficient (<20 ng/ml) N (%)	Insufficient (20–29 ng/ml) N (%)	Sufficient (30–100 ng/ml) N (%)	
Total	134 (46.2)	100 (34.5)	56 (19.3)	290
TB status				
Positive	59 (61.5)	20 (20.8)	17 (17.7)	96
Negative	75 (38.7)	80 (41.2)	39 (20.1)	194
Gender				
Male	56 (39.7)	49 (34.8)	36 (25.5)	141
Female	78 (52.3)	51 (34.2)	20 (13.4)	149
Residence				
Rural	36 (32.7)	42 (38.2)	32 (29.1)	110
Urban	98 (54.4)	58 (32.2)	24 (13.3)	180
Age group				
<15	25 (30.9)	37 (45.7)	19 (23.5)	81
15–24	22 (36.7)	21 (35.0)	17 (28.3)	60
25–34	32 (50.8)	19 (30.2)	12 (19.0)	63
35–44	20 (54.1)	14 (37.8)	3 (8.1)	37
45–54	15 (65.2)	5 (21.7)	3 (13.0)	23
55+	20 (76.9)	4 (15.4)	2 (7.7)	26

*N* number, *TB* tuberculosis

### Risk factors associated with tuberculosis

As shown in Table 3, tuberculosis cases had significantly higher rate of vitamin D deficiency (AOR 3.3; 95% CI 1.8–6.0; p < 0.001) than non TB controls. Significantly higher number of males had active TB compared with females (AOR 2.5; 95% CI 1.4–4.3; p = 0.001). Furthermore, study participants in the age groups of 15–24 (AOR 4.5; 95% CI 2.0–10.2; p < 0.001) and 25–34 (AOR 4.3; 95% CI 1.9–96; p < 0.001) years had significantly higher proportion of active TB compared with age group <15 years of age.

**Table 3:** Risk factors associated with tuberculosis (n = 290)

Characteristics	Total	TB status		Univariate analysis		Multivariate analysis	
		TB patients (N = 96) N (%)	Controls (N = 194) N (%)	COR (95% CI)	p values	AOR (95% CI)	p values
Vit D deficient							
Yes	96	59 (61.5)	37 (38.5)	2.5 (1.5–4.2)	<0.001	3.3 (1.8–6.0)	<0.001
No	194	75 (38.7)	119 (61.3)	1			
Gender							
Male	141	57 (40.4)	84 (59.6)	1.9 (1.2–3.2)	0.010	2.5 (1.4–4.3)	0.001
Female	149	39 (26.2)	110 (73.8)	1			
Residence							
Rural	110	34 (30.9)	76 (69.1)	1			
Urban	180	62 (34.4)	118 (65.6)	1.2 (0.7–2.0)	0.535	0.8 (0.4–1.4)	0.427
Age group							
<15	81	13 (16.0)	68 (84.0)	1			
15–24	60	27 (45.0)	33 (55.0)	4.3 (2.0–9.4)	<0.001	4.5 (2.0–10.2)	<0.001
25–34	63	30 (47.6)	33 (52.4)	4.8 (2.2–10.3)	<0.001	4.3 (1.9–9.6)	<0.001
35–44	37	10 (27.0)	27 (73.0)	1.9 (0.8–4.9)	0.167	1.4 (0.5–3.8)	0.475
45–54	23	6 (26.1)	17 (73.9)	1.8 (0.6–5.6)	0.276	1.3 (0.4–4.2)	0.662
55+	26	10 (38.5)	16 (61.5)	3.3 (1.2–8.8)	0.019	2.0 (0.7–5.6)	0.203

*N* number, *TB* tuberculosis, *COR* crude odds ratio, *AOR* adjusted odds ratio, *Vit D* vitamin D, *CI* confidence interval

### Risk factors associated with vitamin D deficiency

Results of multivariate logistic regression analyses showed that females had significantly higher prevalence of vitamin D deficiency than males (AOR 2.3; 95% CI 1.3–3.9; p = 0.002). Urban residents had significantly higher proportion of vitamin D deficiency compared with rural residents (AOR 3.0; 95% CI 1.7–5.3; p < 0.001). Moreover, study subjects in the age groups of 35–44 (AOR 4.5; 95% CI p = 0.001), 45–54 (AOR 5.8; 95% CI 0 1.8–18.6; p = 0.003) and ≥55 (AOR 7.5; 95% CI 2.3–24.2; p = 0.001) years had significantly higher proportion of vitamin D deficiency compared with age group <15 years of age (Table 4).

**Table 4:** Risk factors associated with vitamin D deficiency (n = 290)

Characteristics	Total	Vit D deficiency		Univariate analysis		Multivariate analysis	
		Deficient (<20 ng/ml) N (%)	Not deficient (20–100 ng/ml) N (%)	COR (95% CI)	p values	AOR (95% CI)	p values
Total	290	134 (46.2)	156 (53.8)				
Gender							
Male	141	56 (39.7)	85 (60.3)	1			
Female	149	78 (52.3)	71 (47.7)	1.7 (1.0–2.7)	0.03	2.3 (1.3–3.9)	0.002
Residence							
Rural	110	36 (32.7)	74 (67.3)	1			
Urban	180	98 (54.4)	82 (45.6)	2.5 (1.5–4.0)	<0.001	3.0 (1.7–5.3)	<0.001
Age group							
<15	81	25 (30.9)	56 (69.1)	1			
15–24	60	22 (36.7)	38 (63.3)	1.5 (0.8–2.9)	0.227	1.0 (0.5–2.1)	0.995
25–34	63	32 (50.8)	31 (45.2)	2.0 (1.0–4.1)	0.054	1.6 (0.7–3.4)	0.252
35–44	37	20 (54.1)	17 (45.9)	3.9 (1.7–9.2)	0.002	4.5 (1.8–11.3)	0.001
45–54	23	15 (65.2)	8 (34.8)	4.9 (1.7–14.2)	0.004	5.8 (1.8–18.6)	0.003
55+	26	20 (76.9)	6 (23.1)	7.6 (2.5–22.9)	<0.001	7.5 (2.3–24.2)	0.001

*N* number, *COR* crude odds ratio, *AOR* adjusted odds ratio, *Vit D* vitamin D, *CI* confidence interval

## Discussion

In this study, a high prevalence of vitamin D deficiency, 46.2% was observed among the total study participants. This finding is in agreement with a previous report from the central part of Ethiopia with the total prevalence of 42% vitamin D deficiency among school children [18]. This finding shows that vitamin D deficiency is highly prevalent among the general community in Ethiopia.

The prevalence of vitamin D deficiency among TB patients (61.5%) reported in our study is comparable to that reported in South Africa (62.7%) [16]. On the contrary, lower prevalence of vitamin D deficiency was reported in Tanzania (10.6%) [8], Guinea Bissau (8.5%) [10] and Uganda (7%) [14] compared to our study. These discrepancies among different reports might be due to the differences in the laboratory assay methods used to measure vitamin D level, definition of vitamin D deficiency, dietary habits of the study population, latitude of the study sites and frequencies of co-morbidities in the study population of different studies. The different techniques used to measure serum vitamin D concentrations in the studies done in Uganda [14], Guinea Bissau [10] and Tanzania [8] were a semi-automated solid phase extraction reverse phase high performance liquid chromatography assay, isotope-dilution liquid chromatography–tandem mass spectrometry on an API3000 mass spectrometer and Radio Immuno Assay (RIA) with ^125^I-labeled 25(OH) D [^125^I-25(OH)D] as tracer using a kit from Immunodiagnostic-Systems, respectively.

In this study, multivariate logistic regression analysis showed a significant association between vitamin D deficiency and tuberculosis (p < 0.001). The possible association between vitamin D deficiency and active tuberculosis was first reported more than 20 years ago [24], however, several studies reported conflicting results. Similar to our study, many studies have reported a significant association between vitamin D deficiency and TB. Studies in West Africa [10], Australia [12], Kenya [17], Vietnam [25], Tanzania [26] and India [27] have reported higher levels of vitamin D deficiency in patients with TB compared with non TB controls. A meta-analysis by Nnoaham et al. also showed that serum vitamin D levels were 0.68 standard deviation lower in TB patients compared to controls [20]. However, studies from Indonesia [28], China [29], Hong Kong [30] and Korea [31] have reported no significant difference in serum vitamin D levels between TB patients and controls. The discrepancies between these studies may be due to differences in cultural characteristics, ethnic, sunlight exposure, skin color or dietary practices. Although there is good evidence to suggest that a decrease in serum vitamin D levels compromises immunity and leads to the re-activation of latent tuberculosis [32], the low serum vitamin D levels may also result from tuberculosis itself.

In the present study, it was noted that a significantly higher level of vitamin D deficiency was observed among females compared with males (p = 0.002). Similarly, a report from Pakistan showed that vitamin D deficiency was significantly higher in females than males [33]. Possible reasons for this female preponderance might be due to poorer nutritional status than their male counterparts, inadequate exposure to sunlight because of the culture of most females to stay at home, and pregnancy experiences.

Increasing age was found to be significantly associated with vitamin D deficiency in our study. Similar findings have also been observed in reports from Uganda [34], and in the USA [35]. Older people are prone to develop vitamin D deficiency because of various risk factors: decreased dietary intake, diminished sunlight exposure, reduced skin thickness, impaired intestinal absorption, and impaired hydroxylation in the liver and kidneys [36–38].

In this study, urban residence was found to be a significant risk factor for vitamin D deficiency compared with rural residence. Our finding was in agreement with the findings of the previous studies in the central part of Ethiopia [18] and in Peru [39] that showed significant association between vitamin D deficiency and urban environment. This might be due to lifestyle changes associated with urbanization that may lead to less time spent outdoors which in turn can be associated with being vitamin D deficient.

The major limitations of our study include dietary intake, biochemical variables (calcium, parathyroid hormone), HIV status and latent TB infections, all of which could affect vitamin D deficiency, were not considered in this study. However, the effect of these factors on the level of vitamin D among TB patients was controlled using tight control groups from the same household who did not have TB diseases. As the cases and controls shared similar environment and household, the cases and controls are likely to have similar risk factors exposures including dietary intake.

## Conclusions

Vitamin D deficiency is highly prevalent among TB patients and non TB controls in Ethiopia where there is year round abundant sunshine. This study confirms a significant association between vitamin D deficiency and tuberculosis in Ethiopia. Vitamin D deficiency was also significantly higher in females, older age groups, and urban residents. The findings of this study warrant further studies first to resolve the chicken–egg dilemma of the association between vitamin D deficiency and TB using cohort study and then to determine whether vitamin D supplementation can have a role in the prevention and treatment of tuberculosis in high TB burden countries like Ethiopia.

## References

[1] World Health Organization (2014). Global tuberculosis report.

[2] Hewison M (2010). Vitamin D and the immune system: new perspectives on an old theme. Endocrinol Metab Clin North Am.

[3] Chocano-Bedoya P, Ronnenberg AG (2009). Vitamin D and tuberculosis. Nutr Rev.

[4] Martineau AR, Wilkinson KA, Newton SM, Floto RA, Norman AW (2007). IFN-gamma- and TNF-independent vitamin D-inducible human suppression of mycobacteria: the role of cathelicidin LL-37. J Immunol.

[5] Ralph AP, Kelly PM, Anstey NM (2008). L-arginine and vitamin D: novel adjunctive immunotherapies in tuberculosis. Trends Microbiol.

[6] Campbell GR, Spector SA (2012). Vitamin D inhibits human immunodeficiency virus type 1 and *Mycobacterium tuberculosis* infection in macrophages through the induction of autophagy. PLoS Pathog.

[7] Liu PT, Stenger S, Li H, Wenzel L, Tan BH (2006). Toll-like receptor triggering of a vitamin D-mediated human antimicrobial response. Science.

[8] Friis H, Range N, Pedersen ML, Mølgaard C, Changalucha J (2008). Hypovitaminosis D is common among pulmonary tuberculosis patients in Tanzania but is not explained by the acute phase response. J Nutr.

[9] Fischer PR, Thacher TD, Pettifor JM (2008). Pediatric vitamin D and calcium nutrition in developing countries. Rev Endocr Metab Disord.

[10] Wejse C, Olesen R, Rabna P, Kaestel P, Gustafson P (2007). Serum 25- hydroxyvitamin D in a West African population of tuberculosis patients and unmatched healthy controls. Am J Clin Nutr.

[11] Holick MF (2007). Vitamin D deficiency. N Engl J Med.

[12] Gibney K, MacGregor L, Leder K (2008). Vitamin D deficiency is associated with tuberculosis and latent tuberculosis infection in immigrants from sub-Saharan Africa. Clin Infect Dis.

[13] van der Meer M, Middelkoop B, Boeke A, Lips P (2011). Prevalence of vitamin D deficiency among Turkish, Moroccan, Indian and sub-Sahara African populations in Europe and their countries of origin: an overview. Osteoporos Int.

[14] Nansera D, Graziano F, Friedman D (2011). Vitamin D and calcium levels in Ugandan adults with human immunodeficiency virus and tuberculosis. Int J Tuberc Lung Dis.

[15] Banda R, Mhemedi B, Allain T (2011). Prevalence of vitamin D deficiency in adult tuberculosis patients at a central hospital in Malawi. Int J Tuberc Lung Dis.

[16] Martineau A, Nhamoyebonded S, Onic T (2011). Reciprocal seasonal variation in vitamin D status and tuberculosis notifications in Cape Town, South Africa. Proc Natl Acad Sci.

[17] Davies P, Church H, Brown R (1987). Raised serum calcium in tuberculosis patients in Africa. Eur J Respir Dis.

[18] Wakayo T, Belachew T, Vatanparast H, Whiting SJ (2015). Vitamin D deficiency and its predictors in a country with thirteen months of sunshine: the case of school children in central Ethiopia. PLoS ONE.

[19] Fogelman Y, Rakover Y, Luboshitzky R (1995). High prevalence of vitamin D deficiency among Ethiopian women immigrants to Israel: exacerbation during pregnancy and lactation. Isr J Med Sci.

[20] Nnoaham KE, Clarke A (2008). Low serum vitamin D levels and tuberculosis: a systematic review and meta-analysis. Int J Epidemiol.

[21] Central Statistical Agency (CSA) of Ethiopia (2008). Summary and statistical report of the 2007 population and housing census.

[22] World Health Organization (2010). Global Tuberculosis Control 2010. WHO/HTM/TB/2010.7..

[23] Moreau E, Bacher S, Mery S, Le Goff C, Piga N, Vogeser M, Hausmann M, Cavalier E (2015). Performance characteristics of the VIDAS^®^ 25-OH Vitamin D Total assay—comparison with four immunoassays and two liquid chromatography-tandem mass spectrometry methods in a multicentric study. Clin Chem Lab Med.

[24] Bartley J (2010). Vitamin D: emerging roles in infection and immunity. Expert Rev Anti Infect Ther.

[25] Ho-Pham LT, Nguyen ND, Nguyen TT (2010). Association between vitamin D insufficiency and tuberculosis in a Vietnamese population. BMC Infect Dis.

[26] Tostmann A, Wielders JPM, Kibiki GS, Verhoef H, Boeree MJ (2010). Serum 25-hydroxy-vitamin D3 concentrations increase during tuberculosis treatment in Tanzania. Int J Tuberc Lung Dis.

[27] Wilkinson RJ, Llewelyn M, Toossi Z (2000). Influence of vitamin D deficiency and vitamin D receptor polymorphisms on tuberculosis among Gujarati Asians in West London: a casecontrol study. Lancet.

[28] Grange JM, Davies PD, Brown RC, Woodhead JS, Kardjito T (1985). A study of vitamin D levels in Indonesian patients with untreated pulmonary tuberculosis. Tubercle.

[29] Chan TY, Poon P, Pang J (1994). A study of calcium and vitamin D metabolism in Chinese patients with pulmonary tuberculosis. J Trop Med Hyg.

[30] Chan TY (1997). Differences in vitamin D status and calcium intake: possible explanations for the regional variations in the prevalence of hypercalcemia in tuberculosis. Calcif Tissue Int.

[31] Koo HK, Lee JS, Jeong YJ (2012). Vitamin D deficiency and changes in serum vitamin D levels with treatment among tuberculosis patients in South Korea. Respirology.

[32] Rook GAW (1988). The role of vitamin D in tuberculosis. Am Rev Respir Dis.

[33] Raheel I, Sultan MK, Adnan Q, Ehtesham H, Hassan BU (2013). Vitamin D deficiency in patients with tuberculosis. J Coll Phys Surg Pak.

[34] Davis K, Edrisa M, Richard S, William W, Harriet MK. Vitamin D deficiency among adult patients with tuberculosis: a cross sectional study from a national referral hospital in Uganda. BMC Res Notes. 2013; 6:293. http://www.biomedcentral.com/1756-0500/6/293. 10.1186/1756-0500-6-293 PMC3737052 23886009

[35] Gloth FM, Gundberg CM, Hollis BW, Haddad JG, Tobin JD (1995). Vitamin D deficiency in homebound elderly persons. JAMA.

[36] Omdahl JL, Garry PJ, Hunsaker LA, Hunt WC, Goodwin JS (1982). Nutritional status in a healthy elderly population: vitamin D. Am J Clin Nutr.

[37] McKenna MJ (1992). Differences in vitamin D status between countries in young adults and the elderly. Am J Med.

[38] Holick MF (1995). Environmental factors that influence the cutaneous production of vitamin D. Am J Clin Nutr.

[39] Checkley W, Robinson CL, Baumann LM (2015). 25-hydroxy vitamin D levels are associated with childhood asthma in a population-based study in Peru. Clin Exp Allergy.

